# Supplementary material for: Genetics of circulating proteins in newborn babies at high risk of type 1 diabetes
Source: Nat Commun. 2025 Apr 22;16:3750. doi: 10.1038/s41467-025-58972-3 (PMC12015297; doi:10.1038/s41467-025-58972-3)
Supplement: Supplementary file 1 — Supplementary Information [file 41467_2025_58972_MOESM1_ESM.pdf]

Genetics of circulating proteins in newborn babies at high risk of type 1 diabetes

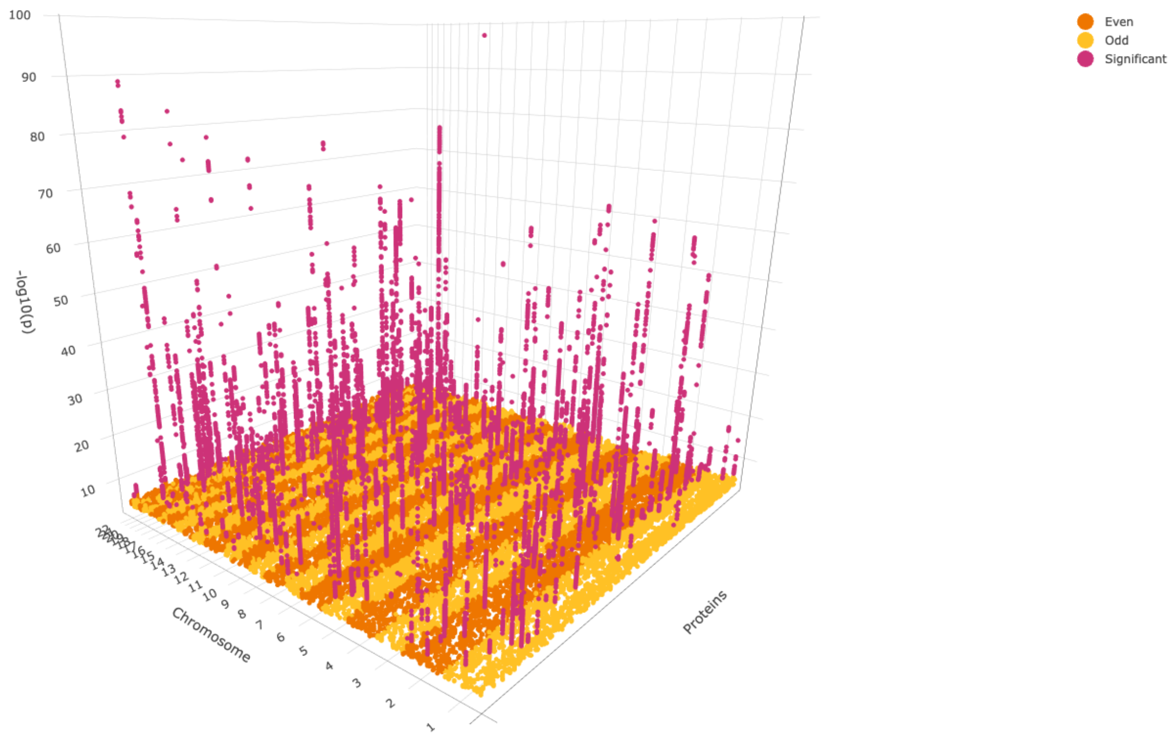

Supplementary Figure 1: 3D Manhattan plot of all tested protein-SNP pairs. The 1985 circulating proteins tested in dried blood spots are represented on the x-axis and sorted alphabetically. The y-axis represents the position of the protein coding gene. The z-axis represents the −log10 p-values.

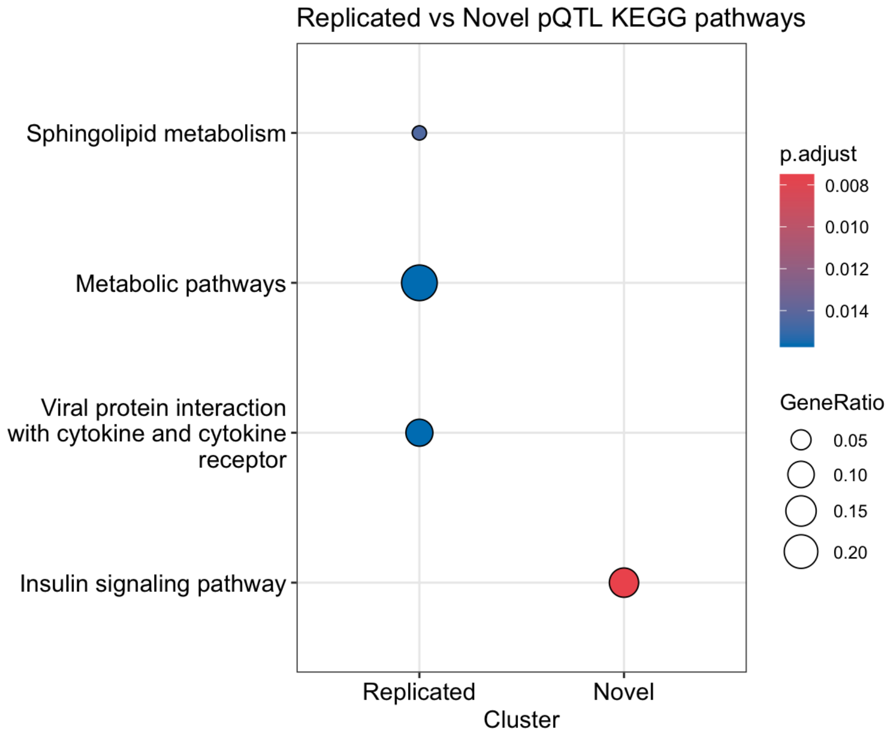

Supplementary Figure 2: Enriched KEGG pathways for the replicated and novel pQTL-targeted proteins. The background protein list consists of the 1985 analyzed proteins. Benjamini–Hochberg was used for p-value correction. The full results are available in the supplementary Data 3. Only results with p.adjust < 0.05 are displayed.

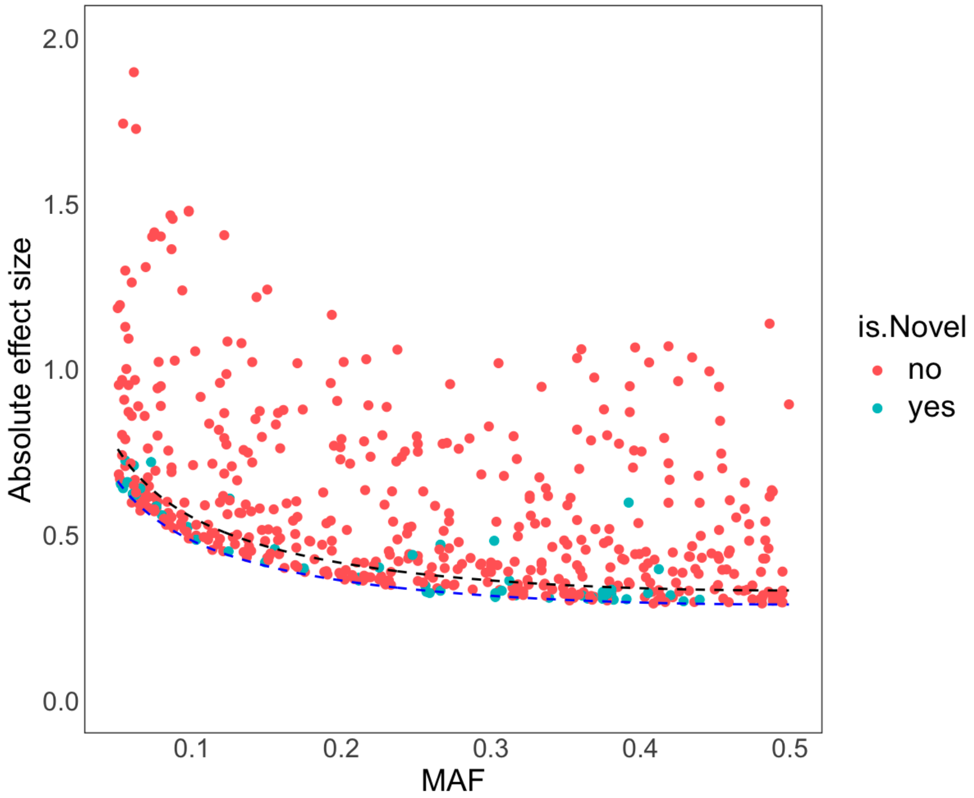

Supplementary Figure 3: Comparison of the minor allele frequency and effect size (beta) for the novel and known pQTL signals. Dashed lines represent the estimated minimum detectable beta for each given MAF with a power of 80% (black), or 50% (blue), with a sample size of 695 and significance threshold 5x10-8 for inverse normal transformed protein levels. The estimated beta has been calculated with the powerEQTL R package.

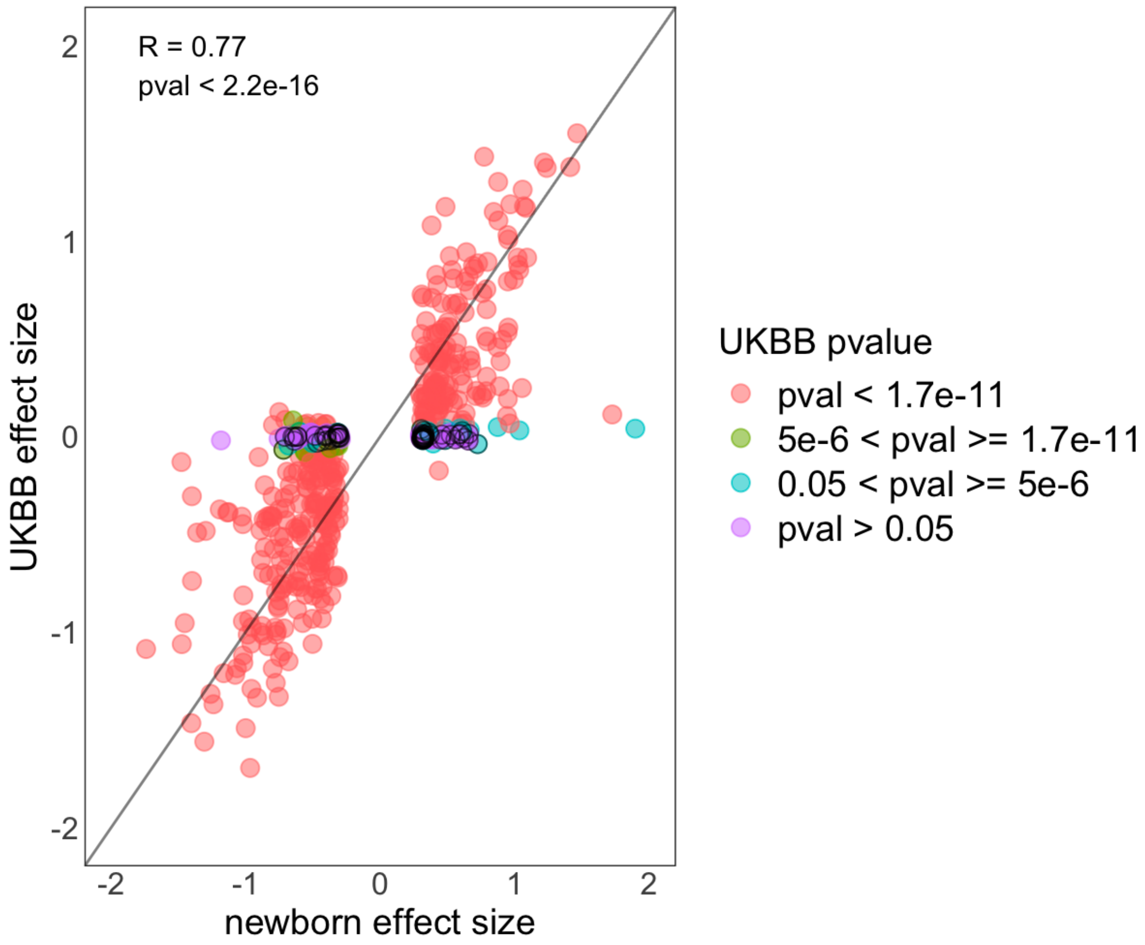

Supplementary Figure 4: Comparison of effect sizes (betas) between newborn and adult pQTL signals. Dots circled in black highlight the signals not previously identified in any of the 46 studies from supplementary table 2 but that are at least nominally significant in the UKBB (Sun et al., Nature, 2023). R=Pearson's correlation coefficient

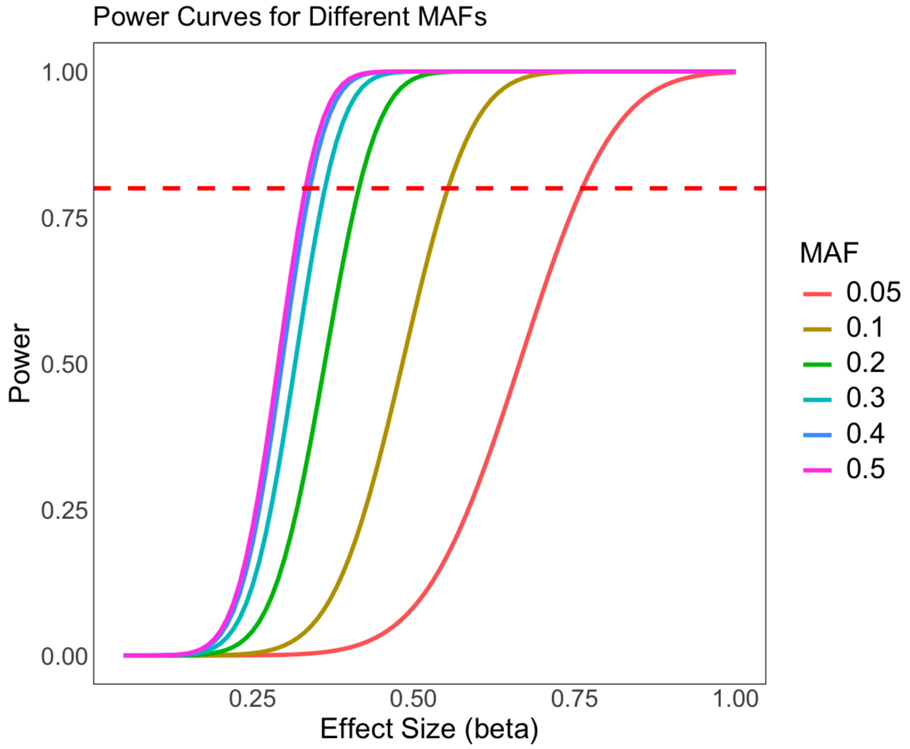

Supplementary Figure 5: Power curves. Power curves for each regression estimate (beta) as a function of minor allele frequency (MAF). The red dashed line denotes power of 80%. The power curves were estimated using the PowerEQTL R package, with a sample size of 695 and significance threshold $5x10^{-8}$ for inverse normal transformed protein levels
