# Supplementary material for: Genetics of circulating proteins in newborn babies at high risk of type 1 diabetes
Source: Nat Commun. 2025 Apr 22;16:3750. doi: 10.1038/s41467-025-58972-3 (PMC12015297; doi:10.1038/s41467-025-58972-3)
Supplement: Supplementary file 2 — Description of Additional Supplementary Files [file 41467_2025_58972_MOESM2_ESM.pdf]

File Name: Supplementary Data 1
Description: *cis*- and *trans*-pQTL signals detected by GCTA-COJO. P-value < 5e-8; The column "Studies" denotes the list of studies (as reported in Supplementary Data 2) in which a pQTL signal for the corresponding protein was found within +/- 1Mb from the newborn pQTL lead SNP reported in the 'SNP (hg19)' column

File Name: Supplementary Data 2
Description: List of studies used to investigate the novelty of the identified pQTLs reported in Supplementary Data 1

File Name: Supplementary Data 3
Description: KEGG enrichment analysis of the novel and replicated pQTL-targeted proteins. Cluster referers to the type of pQTL, either "Replicated" on "Novel". Novelty is defined based on the studies listed in Supplementary Data 2 (n=62)

File Name: Supplementary Data 4
Description: pQTL analysis in the UKBB subset with self-reported T1D (N=61) for the 62 novel pQTLs. Linear regression was used to calculate the genotype association with inverse normal transformed NPX values. Sex, age, mean NPX and time between sample collection (field 3166) and data generation were used as covariates.

File Name: Supplementary Data 5
Description: Results of coloc.fast for *cis*-pQTL signals overlapping T1D regions, +/-1Mb around the GWAS index SNP position.

File Name: Supplementary Data 6
Description: Results of coloc.fast for *trans*-pQTL signals overlapping T1D regions, +/-1Mb around the GWAS index SNP position.

File Name: Supplementary Data 7
Description: Summary statistics of the instrumental variables used in the MR analysis. OR=Odds ratio; eaf= effect allele frequency; se=standard error.

File Name: Supplementary Data 8
Description: STROBE-MR: Mendelian randomization checklist
